# A Case of Ecstasy-Induced Acute Hepatic Injury

**DOI:** 10.7759/cureus.30377

**Published:** 2022-10-17

**Authors:** Nava R Sharma, Bharosa Sharma, Saral Lamichhane, Madalasa Pokhrel, Sudarshan Gautam

**Affiliations:** 1 Medicine, Manipal College of Medical Sciences, Pokhara, NPL; 2 Internal Medicine, John H. Stroger, Jr. Hospital of Cook County, Chicago, USA; 3 Internal Medicine, Gandaki Medical College, Pokhara, NPL; 4 Internal Medicine, Montefiore Medical Center, New Rochelle, USA; 5 Internal Medicine, Maimonides Medical Center, Brooklyn, USA

**Keywords:** substance recreational use, drug-induced acute liver failure, metabolism of mdma, acute liver failure (alf), ecstasy

## Abstract

The recreational use of a drug such as 3,4-methylenedioxymethamphetamine (MDMA), also known as "ecstasy," may be associated with significant side effects. Although liver failure with ecstasy is rare, the use of the drug should be investigated in all patients with severe hepatitis of unknown origin. Early diagnosis and intervention can prevent patients from ending up in liver transplantation. Here, we present a case of a 27-year-old female who developed acute liver injury secondary to recreational intoxication with ecstasy.

## Introduction

The recreational use of a drug such as 3,4-methylenedioxymethamphetamine (MDMA, also known as "ecstasy") is common among young people [1]. It is popular because it can cause disinhibition, euphoria, wakefulness, sexual arousal, and increased alertness and self-esteem [2]. The spectrum of liver injury can range from asymptomatic elevation of liver function tests (LFT) to acute fulminant liver failure requiring liver transplantation. The severity of the damage is accelerated with concomitant consumption of alcohol or other hepatotoxic drugs such as acetaminophen [3]. Ecstasy-induced liver toxicity is usually idiosyncratic and, thus, unpredictable. Here, we present a case of a 27-year-old female with acute liver injury following the use of ecstasy.

## Case presentation

A 27-year-old female presented to our center after stepping over a nail and was incidentally found to have an abnormal liver function test. She had multiple episodes of nausea, vomiting, and abdominal pain before the day of the presentation. She occasionally drank one to two bottles of beer on the weekends. She denied taking alcohol on weekdays. Her last drink was two weeks back. She denied taking any herbal supplements; however, she takes ecstasy drugs almost three pills per week; the last intake was four days ago. The patient was sexually active and monogamous. She denied any family history of liver disease.

On physical examination, her vital signs were normal. The abdomen was soft and non-tender to palpation. She was alert and oriented to time, place, and person on neurological examination, with intact cranial nerves and normal cerebellar testing. There was no evidence of asterixis or any focal neurological deficits.

**Figure 1:**
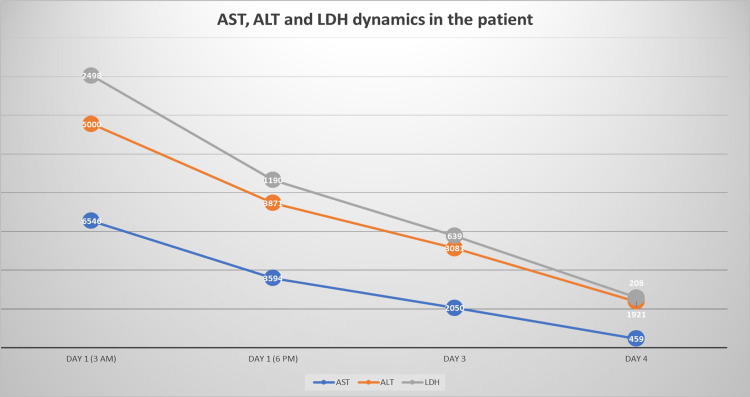
Trend of aspartate transaminase (AST), alanine transaminase (ALT), and lactate dehydrogenase (LDH) during the hospital stay.

**Figure 2:**
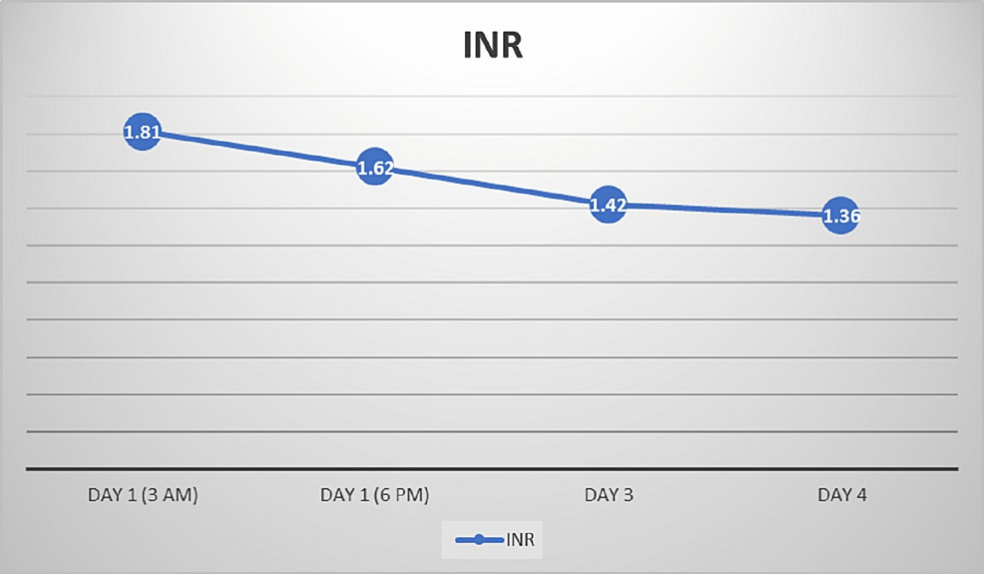
Trend of international normalized ratio (INR) during the hospitalization.

Her initial liver function tests showed aspartate aminotransferase (AST) of 6546 U/L (reference range: 5-40 U/L), alanine aminotransferase (ALT) of >5000 U/L (reference range: <31 U/L), and lactate dehydrogenase (LDH) of 2498 U/L (reference range: 105-333 U/L). Subsequent reports were represented as shown in Figure 1. She had marked elevation of AST and ALT but normal gamma-glutamyl transferase (GGT) and bilirubin levels. That raised an initial concern for rhabdomyolysis and the muscle source of her enzyme abnormalities. A creatine kinase (CK) level was done, which came back as 167 U/L (reference range for females: 30-135 U/L). Her international normalized ratio (INR) on admission was 1.81 (reference range: 0.8-1.2) and subsequently reduced throughout her hospitalization, as shown in Figure 2. Her complete blood count (CBC), random blood sugar, and renal function test were normal. Her viral and autoantibody screens were negative. The liver was of normal dimensions on ultrasound evaluation, but the parenchyma was minimally hypoechoic, granular, and heterogenic. No cholestasis or common bile duct stone was seen.

We diagnosed this as a case of ecstasy-induced acute liver injury after ruling out viral hepatitis, Wilson's disease, hemochromatosis, and autoimmune hepatitis. Pre-existing liver diseases were excluded by clinical history. The history of exposure to ecstasy and clinical improvement after stopping it supported our diagnosis.

We started the patient on N-acetylcysteine and intravenous fluid and regularly monitored her liver function test and coagulation profile. In addition, she was observed for the need to be referred to a transplant center based on King's College Criteria [4]. She was discharged with clear instructions about drug avoidance of Tylenol, alcohol, or any recreational drugs. She was advised to come to the emergency department in case of bleeding, abdominal pain, or confusion noticed by her caregiver. She was followed up after a week with LFT, which had come to a baseline.

## Discussion

MDMA is a synthetic compound with structural properties similar to both amphetamines and mescaline. In 1970, it was first used as a psychotherapeutic agent; before that, it was sometimes used as an appetite reducer [5]. MDMA is typically taken as a tablet in dosages ranging from 50 to 200 mg. It is readily absorbed from the intestinal tract with peak effects that occur within two hours of ingestion and typically last four to six hours. Most MDMA is excreted in the urine, while the hepatic enzyme metabolizes the remainder [6]. Consequently, the concentrations of MDMA in illicitly produced pills vary widely, leading to unpredictable consequences. Although death may occur even after the ingestion of a single tablet, the ingestion of larger quantities carries a greater risk of toxicity.

The exact mechanism of liver injury remains unclear. However, it is supposed to be due to its impaired metabolism, dose-dependent direct toxicity, glutathione level depletion, and systemic hypoperfusion [7]. The liver injury may range in clinical expressions from asymptomatic liver injury to acute hepatic failure. Neither the occurrence nor the severity of hepatocellular toxicity can be predicted, and the severity of symptoms is unrelated to the magnitude or the length of exposure. Fulminant hepatitis has been described after the ingestion of just one tablet of ecstasy. The onset of liver toxicity can range from one day to a few weeks after the ingestion of MDMA. Detecting the drug or its metabolites in biological specimens at the diagnosis usually fails. The clinical course is the most reliable prognostic factor in MDMA-induced hepatitis.

The treatment of this condition mainly consists of supportive measures. Treatment with N-acetylcysteine is often initiated, but evidence from randomized controlled trials is lacking [6]. There is no evidence of the use of corticosteroids or other immunosuppressant drugs. Liver transplantation is an option in severe cases [8]. Molecular Adsorbent Recirculating System (MARS®) therapy or "liver dialysis" has also been tried in a case of acute liver failure in an adolescent male secondary to the recreational use of ethanol and cocaine in addition to MDMA [9].

## Conclusions

Ecstasy-induced acute hepatic failure cases have been increasing in young adults and adolescents. Therefore, physicians should consider this differential in every liver failure case. Although treatment is challenging, early identification and supportive treatment call can reduce the transplant requirement. The exact mechanism of liver injury due to ecstasy remains unclear, and more research is required in this field. Young people should be more aware of ecstasy's side effects.
